# The Science and Art of Surgery

**Published:** 1885-03

**Authors:** 


**The Science and Art of Surgery.** A Treatise on Surgical Injuries, Diseases and Operations. By John Eric Erichsen, F. R. S., LL. D., F. R. C. S., Professor of Surgery and of Clinical Surgery in University College, etc. Eighth edition. Revised and edited by Marcus Beck, M. S., F. R. C. S., Surgeon to University College Hospital. Vol. i. Philadelphia: H. C. Lea's Son & Co. 1884.

Erichsen's surgery needs no introduction from us. It has been a standard work in this country, as well as in Great Britain, for more than thirty years, and has steadily grown in favor with both practitioners and students. The last edition is deserving of all praise. In its preparation the distinguished author has called to his aid the services of distinguished specialists. All that was antequated has been dropped from its pages, and the most recent advances in modern surgery have been incorporated in the work. The first volume contains 1124 pages. The illustrations are abundant, and many of them new. The work, as revised, is deserving of the continued confidence of the profession.

